# Anatomical Analysis and Surgical Correction of Regional Nasal Deformities in Primary Rhinoplasty: A Clinical Observational Study

**DOI:** 10.7759/cureus.88396

**Published:** 2025-07-20

**Authors:** Rajshri Yadav, Heena Masrat, Fozia Wani, Nazir A Khan

**Affiliations:** 1 Otorhinolaryngology, Umanath Singh Autonomous State Medical College, Jaunpur, IND; 2 Otorhinolaryngology, Urban Primary Health Centre, Srinagar, IND; 3 Otorhinolaryngology, Aga Syed Yousuf Memorial Hospital, Watra Wani, IND; 4 Otorhinolaryngology, Government Medical College, Baramulla, Baramulla, IND

**Keywords:** dorsal hump, nasal deformity, nasal subunits, rhinoplasty, septal deviation

## Abstract

Introduction

Rhinoplasty is one of the most intricate procedures in facial plastic surgery, requiring a balance between functional restoration and aesthetic refinement. The existing literature often emphasizes comparisons between surgical techniques; however, there is a lack of studies that systematically evaluate deformity distribution across nasal regions and correlate these findings with corrective strategies. This study aims to analyze the anatomical patterns of nasal deformities in primary rhinoplasty patients, and document the region-specific surgical interventions employed.

Methods

This prospective clinical observational study was conducted at the Department of Otorhinolaryngology, Sher-I-Kashmir Institute of Medical Sciences, Srinagar, India, from August 2013 to December 2015. Thirty patients (n = 17 males and n = 13 females) with primary external nasal deformities were included. Each patient was evaluated for anatomical involvement - upper two-thirds, lower one-third, or both - and intraoperative corrective techniques, including the use and type of autologous grafts, were recorded.

Results

Out of the total 30 patients, 14 subjects (46.67%) had deformities involving both the upper two-thirds and lower one-third of the nose, 10 (33.33%) had isolated lower one-third deformities, and six (20.00%) had upper two-thirds involvement only. The most frequent deformity was dorsal hump (n = 24 patients, 80.00%), followed by dorsal deviation (n = 18 patients, 60.00%), reduced columellar support (n = 10 patients, 41.67%), and caudal septal deviation (n = 5 patients, 20.83%). Autologous cartilage grafts were used in 15 patients (50.00%), with septal cartilage used in 13 (86.67%), and conchal cartilage in two subjects (13.33%).

Conclusion

Region-based anatomical analysis is primary for planning primary rhinoplasty. Customized surgical correction, supported by targeted use of autologous grafts, improves structural integrity and enhances aesthetic outcomes.

## Introduction

The nose is a central midline facial structure that plays a critical role not only in breathing and olfaction but also in overall facial aesthetics. Given its prominent location, even minor nasal deformities can result in significant cosmetic and functional concerns [1]. These deformities may be congenital, developmental, or acquired - most commonly due to trauma - and often present as external contour irregularities, internal structural deviations, or a combination of both. For optimal surgical outcomes, precise identification of the anatomical region involved is essential [2].

Rhinoplasty is a procedure in which millimeter-level adjustments can lead to substantial aesthetic and functional improvements [3]. A comprehensive understanding of nasal anatomy is crucial, particularly the division of the nose into aesthetic subunits and segments. It includes nine key subunits - the tip, dorsum, alar base, and columella - and six aesthetic segments (the dorsal, lateral wall, and columellar segments) [4]. This anatomical classification helps the surgeon to map deformities with precision and design correction strategies for an individual person. Three major nasal angles, such as the nasofrontal, nasofacial, and nasolabial, are reference points in the aesthetic evaluation of the nasal framework [5]. Deviations in these angles often signal region-specific abnormalities, such as dorsal humps, columellar retraction, or under-projected nasal tips. Understanding these angles in relation to ethnic and gender norms is essential for guiding surgical planning and postoperative assessment [6].

The choice of surgical method must be determined by the specific anatomical region involved. Additionally, deformities in the upper two-thirds of the nose, involving the bony and cartilaginous vault, may require techniques such as dorsal hump reduction, osteotomies, or spreader graft placement [7]. In contrast, lower one-third deformities, involving the tip and columella, are often corrected using shield grafts, columellar struts, or the Tongue-in-Groove (TIG) technique for tip projection and rotation [8]. Moreover, augmentation rhinoplasty is employed in cases of saddle nose or depressed dorsum, whereas reduction rhinoplasty techniques are applied in cases of dorsal convexity or bulky alar cartilages [5]. Cartilage grafting is important in restoring aesthetics as well as structural integrity. Autologous grafts, typically harvested from the nasal septum or conchal bowl, are favored for their excellent biocompatibility, minimal risk of extrusion, and durable long-term results. When strategically applied according to the specific anatomical region of deformity, these grafts significantly improve surgical outcomes and reduce the likelihood of revision procedures [9].

Despite extensive literature on rhinoplasty techniques, few studies offer a detailed analysis of the anatomical distribution of nasal deformities and their corresponding region-specific surgical corrections in primary cases. This study aims to fill that gap by providing a systematic evaluation of the anatomical patterns underlying nasal deformities and their correlation with individualized surgical approaches. The objective is to establish a clinically relevant framework that enhances diagnostic accuracy and promotes tailored surgical planning, thereby improving consistency and patient satisfaction in primary rhinoplasty outcomes.

## Materials and methods

Study design and duration

This prospective observational study was conducted in the Department of Otorhinolaryngology, Head and Neck Surgery, at Sher-I-Kashmir Institute of Medical Sciences, Srinagar, India, over a period of 29 months, from August 2013 to December 2015. The study was approved by the Institutional Ethics Committee and follows the Declaration of Helsinki (approval no. SKIMS/MC/CM/IEC/19). The primary aim was to analyze the anatomical regions involved in external nasal deformities in patients undergoing primary rhinoplasty, and to evaluate the specific surgical corrections employed based on the region and type of deformity.

Participants and selection criteria

Thirty patients presenting with external nasal deformities were prospectively enrolled after satisfying predefined eligibility criteria. All patients were undergoing primary rhinoplasty, with deformities requiring anatomical correction involving the bony vault, cartilaginous dorsum, nasal tip, columella, or alar base. The inclusion criteria consisted of patients aged 15 to 55 years, of either sex, with clinically evident external nasal deformities suitable for surgical correction, and no prior history of nasal surgery. The exclusion criteria included patients with a history of revision rhinoplasty, systemic illnesses, facial growth abnormalities, chronic granulomatous diseases, or those with unrealistic expectations or unwillingness to provide informed consent.

The selection of surgical technique - open versus closed approach - was based on clinical assessment of the type, extent, and anatomical location of the deformity. Patients with complex, multi-regional deformities, particularly those involving the lower one-third or requiring extensive structural correction, were managed with the open approach. Conversely, patients with more localized or less severe deformities were treated using the closed technique. This method of approach selection reflects standard surgical decision-making and was not randomized.

Preoperative assessment and anatomical classification

Each patient underwent a comprehensive clinical and photographic assessment to classify the anatomical location and type of nasal deformity. Standardized photographs were captured in four views: frontal, lateral, basal, and oblique, and analyzed for nasal symmetry, contour, and deviation. Deformities were grouped into three anatomical regions based on involvement of aesthetic nasal subunits: (1) upper two-thirds (bony dorsum and upper lateral cartilages), (2) lower one-third (tip, alar cartilages, columella), and (3) combined upper and lower one-third deformities. Within these regions, specific morphological abnormalities - such as dorsal hump, dorsal deviation, wide bony vault, lateral wall depression, under-projected tip, columellar retraction, and caudal septal deviation - were identified and documented. Photographic analysis was also used to measure aesthetic nasal angles, including nasofrontal, nasofacial, and nasolabial angles, providing a quantitative assessment of nasal proportions. Clinical evaluation included both static (at rest) and dynamic (during inspiration) assessments. Static assessment involved visual inspection and palpation to evaluate surface irregularities and cartilage integrity. In contrast, dynamic assessment - including Cottle’s test - was used to detect nasal valve collapse and identify structurally unsupported or compromised regions during nasal airflow. These combined methods ensured a detailed anatomical mapping of deformities, guiding region-specific surgical planning.

Surgical strategy and region-based correction

Surgical planning was individualized according to the region and nature of the anatomical deformity. Although both open and closed approaches were employed, this study did not analyze outcomes based on the surgical approach. Instead, the focus was on anatomically directed corrections. Deformities of the upper two-thirds were addressed using dorsal hump reduction, osteotomies for realignment of the nasal pyramid, spreader grafts to correct dorsal irregularity or collapse, and augmentation in cases of saddle nose. In patients with lower one-third deformities, tip modification procedures were employed, including columellar strut placement, shield grafts, and the TIG technique for tip projection and stabilization. Patients with combined deformities underwent staged corrections, targeting each involved region.

Cartilage grafting

Autologous cartilage grafts were used in patients requiring structural reinforcement. Septal cartilage was the preferred source due to its availability and stability, and it was used in the majority of grafting cases. In patients with inadequate septal cartilage, conchal cartilage was harvested and shaped intraoperatively. Grafts were used for dorsal augmentation, internal valve stabilization, columellar support, and contour enhancement of the nasal tip.

Postoperative follow-up and outcome evaluation

Patients were followed for a period of 7 to 12 months. Postoperative evaluations focused on both the aesthetic correction of the anatomical deformity and the integration of grafts. Follow-up photography was performed at regular intervals to compare preoperative and postoperative anatomical alignment, nasal angles, and contour symmetry. Patients were assessed for residual deformity, graft-related complications, and overall satisfaction.

Statistical analysis

All clinical data, anatomical classifications, and intraoperative findings were recorded systematically. Categorical data, such as region involved, deformity type, and graft usage, were expressed in frequencies and percentages. Nasal angle measurements and follow-up observations were compiled for descriptive analysis. Statistical testing was not the primary objective, as the study focused on descriptive and anatomical mapping rather than comparative or inferential outcomes.

## Results

Patient characteristics

The study included 30 patients who underwent primary rhinoplasty for external nasal deformities. Of these, 17 (56.7%) were males and 13 (43.3%) were females, with a male-to-female ratio of 1.3:1. The ages of the patients ranged from 15 to 55 years, with a mean age of 29.8 years. The most common presenting complaint was a combination of external nasal deformity with nasal obstruction, reported in 21 patients (70%), while nine patients (30%) presented with aesthetic deformity alone (Table 1).

**Table 1:** Baseline characteristics

Variables	Number of patients (n)	Percentage (%)/range
Total number of patients	30	100
Sex		
Male	17	56.67
Female	13	43.33
Age (in years)		
Range		15 - 55 years
Mean age		29.8 years
Presenting complaints		
Aesthetic deformity only	9	30
Aesthetic + nasal obstruction	21	70

Anatomical distribution of nasal deformities

Analysis of the anatomical involvement revealed that deformities affecting both the upper two-thirds and lower one-third of the nose were the most frequent, seen in 14 patients (46.67%). Isolated involvement of the lower one-third was noted in 10 patients (33.33%), while six patients (20.00%) had deformities confined to the upper two-thirds of the nose (Table 2).

**Table 2:** Anatomical region involved in nasal deformity

Region involved	Number of patients (n)	Percentage (%)
Upper two-thirds only	6	20.00
Lower one-third only	10	33.33
Both upper two-thirds and lower one-third	14	46.67

Specific deformities by anatomical region

An anatomical assessment of nasal deformities was performed to see the involvement of the upper two-thirds and lower one-third of the nose. Among the 30 patients included in the study, deformities involving the upper two-thirds (i.e., bony vault and upper lateral cartilages) were observed in 20 patients (66.67%), including both isolated and combined cases. The most commonly encountered deformity in this region was a dorsal hump, present in 16 patients (80.00%). This was followed by dorsal deviation in 12 patients (60.00%), wide bony vault in five patients (25.00%), and lateral wall depression in four patients (20.00%) (Table 3).

**Table 3:** Deformities in the upper two-thirds of the nose (n = 20)

Type of deformity	Number of patients	Percentage (%)
Dorsal hump	16	80.00
Dorsal deviation	12	60.00
Wide bony vault	5	25.00
Lateral wall depression	4	20.00

Involvement of the lower one-third of the nose (including the nasal tip, alar cartilages, and columella) was noted in 24 patients (80.00%), again including both isolated and combined cases. The most frequent deformity in this region was reduced columellar support, observed in 10 patients (41.67%), followed by under-projected tip and caudal septal deviation, each seen in five patients (20.83%). As with the upper third, multiple deformities were often observed in individual patients, so the cumulative total exceeds 24 patients (80.00%) (Table 4).

**Table 4:** Deformities in the lower one-third of the nose (n = 24)

Type of deformity	Number of patients	Percentage (%)
Reduced columellar support	10	41.67
Under-projected tip	5	20.83
Caudal septal deviation	5	20.83

Surgical corrections based on region and deformity

All surgical corrections were tailored to the anatomical region and specific deformity identified. Among the patients with dorsal hump or dorsal deviation, hump reduction and osteotomies were routinely performed. In cases of dorsal deviation or collapse, spreader grafts were used primarily to address dorsal deviation and internal valve collapse; in addition, they served to reinforce the middle vault, maintain dorsal aesthetic lines, and prevent postoperative inverted V deformity. Four patients received spreader grafts, of which three were fashioned from autologous septal cartilage and one from conchal cartilage. For lower one-third deformities, the most frequently employed technique was columellar strut placement using the TIG technique, used in 15 patients (50%). Thirteen of these used septal cartilage, and two used conchal cartilage. Shield grafts were used in one patient for tip refinement, and septal extension grafts were used in two cases to enhance tip projection and correct caudal septal deviation (Table 5).

**Table 5:** Region-specific surgical techniques and graft usage TIG, Tongue-in-Groove

Surgical technique	Number of patients	Graft type used
Spreader graft	4	3 septal, 1 conchal
Columellar strut (TIG technique)	15	13 septal, 2 conchal
Septal extension graft	2	2 septal
Shield graft (tip refinement)	1	1 conchal
Dorsal augmentation (saddle nose)	1	1 conchal

Postoperative complications

Postoperative complications were observed in a limited number of patients who underwent region-specific anatomical correction with autologous grafting. Among the 15 graft recipients, mild persistence of nasal deviation was noted in two patients (13.33%), while columellar retraction occurred in one patient (6.67%). Importantly, no patients developed persistent nasal obstruction or intranasal synechiae during follow-up. All complications were minor in nature and managed conservatively, with no requirement for revision surgery (Table 6).

**Table 6:** Postoperative complications in patients with grafts (n = 15)

Complication	Number of patients	Percentage (%)
Mild persistence of deviation	2	13.33
Columellar retraction	1	6.67
Persistent nasal obstruction	0	0.00
Synechiae formation	0	0.00

## Discussion

The present study aimed to evaluate the anatomical patterns of nasal deformities in primary rhinoplasty and to assess region-specific surgical corrections using autologous grafts. The analysis revealed a high frequency of complex deformities involving both functional and aesthetic components, requiring individualized surgical planning. Among the 30 patients evaluated, deformities involving both the upper two-thirds and the lower one-third of the nose were most common (n = 14, 46.67%), followed by deformities confined to the lower one-third (n = 10, 33.33%) and the upper two-thirds (n = 6, 20.00%). This distribution reflects the multifocal nature of nasal deformities in primary rhinoplasty, particularly in post-traumatic and developmental cases. These findings are comparable to previous observations in similar anatomical studies [10-12].

In the upper two-thirds region (n = 20), the most frequent deformity was a dorsal hump, observed in 16 patients (80.00%). Dorsal deviation was noted in 12 patients (60.00%), a wide bony vault in five patients (25.00%), and lateral wall depression in four patients (20.00%). These results closely parallel the findings of Foda, who reported dorsal humps in 72% and dorsal deviation in 47% of their large cohort of 500 rhinoplasty cases [10]. The anatomical complexity of the bony vault, especially in post-traumatic noses, has also been emphasized by Teichgraeber et al. [13] and Gubisch and Constantinescu [14], reinforcing the need for precise osteotomies and spreader graft placement for dorsal stabilization. In the lower one-third region (n = 24), reduced columellar support was seen in 10 patients (41.67%), while under-projected tips and caudal septal deviations were each present in five patients (20.83%) [13,14]. These deformities directly affect tip support, projection, and rotation, necessitating reconstruction with columellar struts, septal extension grafts, or shield grafts. Foda similarly highlighted the high incidence of tip deformities requiring structural grafting [10]. The TIG technique, used in 15 patients in our study (50.00%), provided predictable outcomes in restoring tip support and alignment. Berger et al. have also advocated the role of TIG and septal grafts in achieving durable aesthetic corrections [15].

Autologous cartilage grafting was utilized in 15 patients (50.00%), with septal cartilage used in 13 patients (86.67% of graft cases), and conchal cartilage in two patients (13.33%). The preference for septal cartilage is well supported in the literature due to its structural integrity and ease of harvest. Gendeh concluded that autografts, especially septal, carry a low risk of extrusion, resorption, or immune reaction compared to alloplasts, provided proper harvesting and shaping techniques are used [12]. Postoperative graft integration was excellent. Of the 15 patients receiving grafts, 14 (93.33%) demonstrated complete graft take-up without complication, and only one patient (6.67%) developed a minor retraction at the columella, which did not require revision. No cases of graft resorption, infection, or extrusion were noted. These outcomes support the findings of Gendeh [12] and Gubisch and Constantinescu [14], who reported long-term success with structural autografts in primary rhinoplasty. Minor postoperative complications were observed in the broader cohort of 30 patients, including mild persistence of deviation (n = 4, 13.33%), columellar retraction (n = 2, 6.67%), persistent nasal obstruction (n = 3, 10.00%), and synechiae formation (n = 2, 6.67%). All were managed conservatively and did not result in secondary surgical interventions.

This anatomical analysis reinforces that detailed regional assessment of nasal deformities, combined with targeted correction using structural autologous grafts, leads to reliable functional and aesthetic outcomes. The use of septal and conchal cartilage, tailored to the defect and subunit involved, proved highly effective, with minimal morbidity. These results support a structure-based philosophy in primary rhinoplasty, with anatomical mapping serving as a cornerstone in surgical planning.

## Conclusions

Anatomical analysis of nasal deformities is essential for achieving optimal outcomes in primary rhinoplasty. This study highlights that most patients present with complex, multi-regional deformities requiring personalized, region-specific surgical correction. The use of autologous grafts, especially septal and conchal cartilage, proved effective for structural support and aesthetic refinement, with high rates of graft integration and low complication rates. A systematic, anatomy-based approach to surgical planning enhances both functional and aesthetic results, reinforcing the value of individualized treatment strategies in rhinoplasty.
